# *CNTN6* copy number variations in 14 patients: a possible candidate gene for neurodevelopmental and neuropsychiatric disorders

**DOI:** 10.1186/s11689-015-9122-9

**Published:** 2015-08-06

**Authors:** Jie Hu, Jun Liao, Malini Sathanoori, Sally Kochmar, Jessica Sebastian, Svetlana A. Yatsenko, Urvashi Surti

**Affiliations:** Pittsburgh Cytogenetics Laboratory, Center of Medical Genetics and Genomics, Magee-Womens Hospital of UPMC, Pittsburgh, PA 15213 USA; Department of Obstetrics, Gynecology & Reproductive Sciences, University of Pittsburgh School of Medicine, Pittsburgh, PA 15213 USA; Children’s Hospital of Pittsburgh of UPMC, Pittsburgh, PA 15224 USA; Department of Pathology, University of Pittsburgh School of Medicine, Pittsburgh, PA 15213 USA

**Keywords:** 3p26.3 CNV, Array CGH, *CNTNs*, *CNTN6*, Microdeletion, Microduplication, Neurodevelopmental disorders, Neuropsychiatric disorders

## Abstract

**Background:**

Neurodevelopmental disorders are impairments of brain function that affect emotion, learning, and memory. Copy number variations of contactin genes (*CNTNs*), including *CNTN3*, *CNTN4*, *CNTN5*, and *CNTN6*, have been suggested to be associated with these disorders. However, phenotypes have been reported in only a handful of patients with copy number variations involving *CNTNs*.

**Methods:**

From January 2009 to January 2013, 3724 patients ascertained through the University of Pittsburgh Medical Center were referred to our laboratory for clinical array comparative genomic hybridization testing. We screened this cohort of patients to identify individuals with the 3p26.3 copy number variations involving the *CNTN6* gene, and then retrospectively reviewed the clinical information and family history of these patients to determine the association between the 3p26.3 copy number variations and neurodevelopmental disorders.

**Results:**

Fourteen of the 3724 patients had 3p26.3 copy number variations involving the *CNTN6* gene. Thirteen of the 14 patients with these *CNTN6* copy number variations presented with various neurodevelopmental disorders including developmental delay, autistic spectrum disorders, seizures and attention deficit hyperactivity disorder. Family history was available for 13 of the 14 patients. Twelve of the thirteen families have multiple members with neurodevelopmental and neuropsychiatric disorders including attention deficit hyperactivity disorder, seizures, autism spectrum disorder, intellectual disability, schizophrenia, depression, anxiety, learning disability, and bipolar disorder.

**Conclusions:**

Our findings suggest that deletion or duplication of the *CNTN6* gene is associated with a wide spectrum of neurodevelopmental behavioral disorders.

## Background

Contactins (CNTNs) are members of a protein subfamily of neural immunoglobulin (Ig) domain-containing cell adhesion molecules, which may play a role in the formation of axon connections in the developing nervous system. The *CNTN4* and *CNTN6* genes are located at the distal part of the short arm of chromosome 3 (3p26.3). The *CNTN4* gene has been proposed as one of the critical genes for chromosome 3pter-p25 deletion syndrome [1]. The characteristic features associated with this syndrome include prenatal and postnatal growth retardation, developmental delay (DD), intellectual disabilities (ID), hypotonia, and microcephaly (OMIM #613792). Gene association studies have shown the involvement of contactin genes (*CNTNs*) in autism spectrum disorders (ASDs) [2, 3]. The disruption of a single copy of the *CNTN4* by a de novo balanced translocation has been reported in a boy with some features of 3p deletion syndrome including DD, mild ID, hypotonia, ptosis, and ASD [4, 5]. In addition, chromosome 3 copy number variations (CNVs) involving the *CNTN4* gene have been reported to be associated with ASD in a few patients without any other classic 3p deletion syndrome phenotype in three independent studies using genome-wide SNP genotyping or microarray analysis [6–8]. The *CNTN6* gene, which encodes another member of the contactin family, mapped just distal to the *CNTN4* gene is also deleted in the 3p deletion syndrome. A study by Cui et al. [9] shows that the CNTN6 (NB-3) participates in the generation of oligodendrocytes by acting as a ligand of NOTCH1 to promote NOTCH1 activation through the released notch intracellular domain (NICD) and subsequent translocation to the nucleus. The expression of *Cntn6* in the mouse brain is at a low level during embryogenesis but increases significantly after birth [10]. Northern blot analysis showed that the level of *CNTN6* expression in human adult brain was the highest in the cerebellum, followed by the thalamus and subthalamic nucleus, and was lower in the corpus callosum, caudate nucleus, and spinal cord [11]. Moreover, studies have shown that *Cntn6*^*−/−*^ deficient mice have impaired motor coordination and abnormal apical dendrite projections of deep layer pyramidal neurons in the visual cortex [12, 13]. In addition, a recent study indicated that CNTN4, CNTN5, and CNTN6 proteins may be a part of the heteromeric receptor complexes and serve as ligands themselves [14]. Therefore, deletion or duplication of the *CNTN6* gene may affect the function of the receptor complex and cause malfunction of the brain and nervous system. These CNVs involving the *CNTN5* or *CNTN6* gene alone have also been reported in a handful of patients with either ASD or ID or DD [15–18]. However, this remains controversial. A study involving a single family concluded that the deletion of the *CNTN6* gene is not associated with dysmorphic features and ID [19]. In this study, we report 3p26.3 CNVs encompassing the *CNTN6* gene in 14 patients. Thirteen of the 14 patients have neurodevelopmental disorders (NDDs) and seven of the 14 patients have dysmorphic features.

## Methods

### Patient ascertainment

From January 2009 to January 2013, 3724 patients ascertained through the University of Pittsburgh Medical Center (UPMC) were referred to our laboratory for clinical array comparative genomic hybridization (aCGH) testing because of the presence of multiple congenital anomalies, heart defect, short stature, DD, ID, ASD, seizures (SZs), or other unexplained anomalies. The clinical information and family history of the patients with the 3p26.3 CNV were retrospectively reviewed. This study was approved by the University of Pittsburgh Institutional Review Board (IRB#: PRO13090288).

### Array-based comparative genomic hybridization analysis

Oligonucleotide-based whole-genome aCGH was performed using a NimbleGen 135K oligonucleotide array, SignatureChip Oligo Solution version 2.0, which was custom-designed by Signature Genomic Laboratories (Spokane, WA, USA) and made by Roche NimbleGen (Madison, WI, USA) as previously described [20]. Results were displayed by custom aCGH analysis software Genoglyphix version 2.6 (Signature Genomic Laboratories).

### Chromosome analysis

High-resolution G-banded chromosome analysis was performed on the peripheral blood specimen following routine protocols. A minimum of 20 metaphase cells were examined for both numerical and structural chromosomal anomalies, and two or more karyograms were created using the Ikaros Karyotyping System (MetaSystems, Waltham, MA, USA) on each patient.

### Fluorescence in situ hybridization analysis

Fluorescence in situ hybridization (FISH) analysis was performed on metaphase spreads of cultured peripheral blood lymphocytes from patients and their parents using standard procedures. FISH probes were made from the RPCI-11 human genomic library (Invitrogen, Carlsbad, CA, USA) using Nick Translation Kit (Abbott Molecular Inc., Des Plaines, IL, USA). Images were captured using the Isis FISH Imaging System v5.3 software (MetaSystems, Waltham, MA, USA).

## Results

**Fig. 1:**
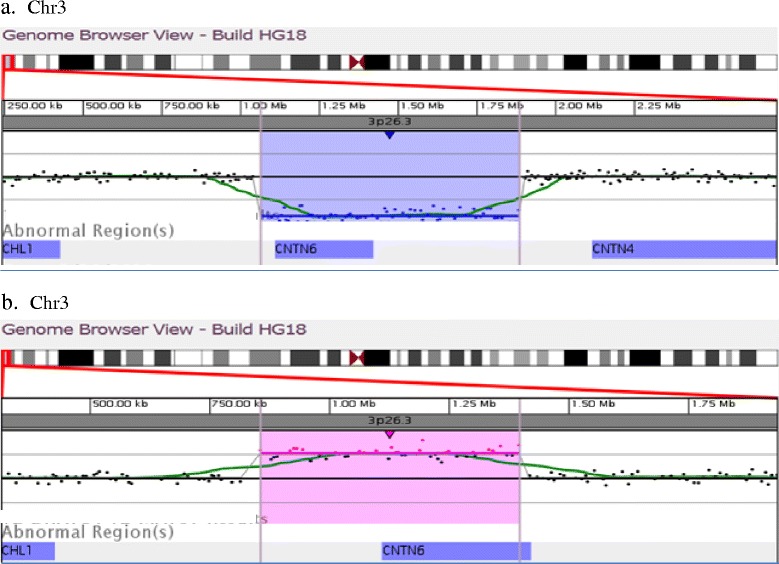
**a** aCGH profile showing an approximately 821-kb deletion in the 3p26.3 region (1,063,289–1,884,842) detected in patient 2; genome browser [hg18] showing the *CNTN6* in the deleted region. **b** aCGH profile showing an approximately 541-kb duplication in the 3p26.3 region (855,662–1,397,384) detected in patient 9; genome browser [hg18] showing *CNTN6* in the duplicated region

**Fig. 2:**
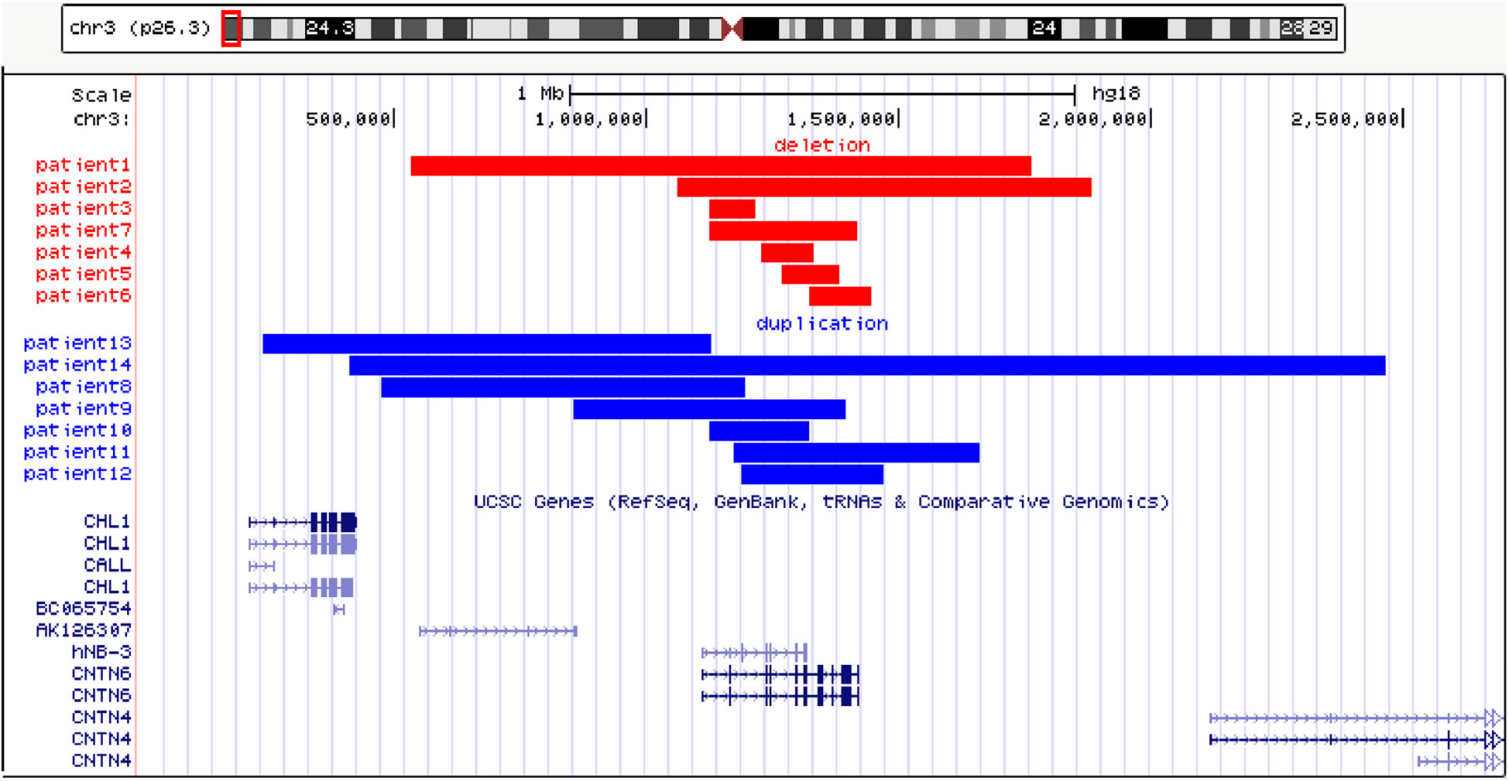
Schematic representation of the array-CGH results of the 14 patients with deletion or duplication of the 3p26.3 region. The ideogram of chromosome 3 and the 3p26.3 highlighted in a *small red box* is shown on *top*. The *red bars* represent deletion sizes, and the *blue bars* represent duplication sizes. The genes in the interval are shown at the *bottom*

Fourteen of the 3724 patients (0.4 %) were found to have CNVs involving the *CNTN6* gene. Seven of the 14 patients had single copy loss in the 3p26.3 region involving deletion of the entire *CNTN6* gene (patients 1 and 2) or intragenic *CNTN6* deletion (patients 3–7). Five of the 14 patients had a single copy gain in the 3p26.3 region involving an intragenic duplication of the *CNTN6* gene (patients 8–12). The remaining two patients (patients 13 and 14) had duplications encompassing *CHL1/CNTN6* and *CHL1/CNTN6/CNTN4* genes, respectively. The representative aCGH profiles (patient 2 and patient 9) are shown in Fig. 1a, b. The schematic representation of the array-CGH results in 14 patients is shown in Fig. 2. The size of the deletions/duplications involving the *CNTN6* gene alone ranged from 93.95 kb to 1.23 Mb. No other known pathogenic CNVs or CNVs with unclear clinical significance were observed in any of these patients. The laboratory and clinical findings of these patients and their family histories are summarized in Tables 1 and 2. The genomic positions of the deletion or the duplication for each patient are illustrated in Fig. 2 and summarized in Tables 1 and 2. The karyotype was normal in all 14 patients. Parental FISH analysis was offered to the families. However, only four families completed the FISH testing (family 6, 8, 11, and 13). The results of the parental FISH analysis indicated that these CNVs are inherited.

**Table 1:** Laboratory findings, clinical features, and family histories of seven patients with 3p26.3 deletion involving *CNTN6* gene

Patients	Age (year)	CNV sizes	Coordinates (hg18)	Inheritance	OMIM genes	Types of deletion	Reasons for referral	Other features	Family history of NDDs and/or neuropsychiatric disorders
1	5	1.23 Mb	535,618–1,763,192	Unknown	*CNTN6*	Whole gene	ASD, DD	Tantrums, aggression	Father: LD; mother: SZs, hearing loss, and mental health issue
2	3	821.55 kb	1,063,289–1,88,4842	Unknown	*CNTN6*	Whole gene	DD, SZs	Nystagmus, macrocephaly, frontal bossing, down-slanting palpebral fissures, high-arched palate	Paternal aunt: ID; maternal aunt, cousin, grandmother, and grandaunt: SZs
3	11	93.95 kb	1,124,286–1,218,241	Unknown	*CNTN6*	Exon 2	DD, SZs, ID	Strabismus, regression in skills, headache	Father: LD; mother: SZs, schizophrenia, migraines; multiple members of maternal side: LD, ASD, SZs, depression, anxiety, bipolar disorder, schizophrenia
4	6	106.77 kb	1,227,323–1,334,091	Unknown	*CNTN6*	Exons 3–7	DD, ASD	Tantrums	Maternal grandmother: depression
5	2	115.87 kb	1,266,963–1,382,828	Unknown	*CNTN6*	Exons 5–12	DD, SZs	Schizencephaly, hydrocephaly, hydronephrosis, diabetes insipidus, hypothyroidism, right-sided spasticity and hemiparesis, cardiomyopathy	Adopted into family; adopted sibling: schizophrenia
6	6	125.24 kb	1,322,292–1,447,530	Paternally inherited	*CNTN6*	Exons 8–23	SZs	Abnormal EEG	Father: del. *CNTN6*, normal phenotype; sister: del. *CNTN6*, SZs; mother and brother: without del. *CNTN6*, phenotypically normal; a maternal aunt: SZs
7	1	244 kb	1,124,286–1,419,226	Unknown	*CNTN6*	Exons 2–23	Heart block	No	No

*ASD* autism spectrum disorder, *CNV* copy number variation, *del.* deletion, *DD* developmental delay, *EEG* electroencephalogram, *ID* intellectual disability, *LD* learning disability, *NDD* neurodevelopmental disorder, *SZs* seizures

**Table 2:** Laboratory findings, clinical features, and family histories of seven patients with 3p26.3 duplication involving *CNTN6* gene

Patients	Age (year)	CNV sizes	Coordinates (hg18)	Inheritance	OMIM genes	Types of deletion	Reasons for referral	Other features	Family history of NDDs and/or neuropsychiatric disorders
8	7	718.82 kb	476,636–1,195,459	Maternally inherited	*CNTN6*	Upstream and exons 1–2	DD, SZs	ADHD, DBD, abnormal EEG, macrocephaly, epicanthal folds, high and wide nasal bridge, broad nasal tip, large central incisors	Mother: normal phenotype; father: schizophrenia, alcoholic; sister: DD; maternal half brother: ADHD, hearing loss, anxiety
9	8	541.62 kb	855,662–1,397,284	Unknown	*CNTN6*	Upstream and exons 1–17	DD	Short stature, reflux	Father: depression; mother: ADHD, anxiety
10	6	199.60 kb	1,124,286–1,323,884	Unknown	*CNTN6*	Exons 2–7	DD	Short stature, reflux, joint hypermobility, microcephaly, low anterior hairline, second and third toe syndactyly	A sister: ASD, microcephaly; another sister: microcephaly; brother: ASD; paternal half brother: ADHD; both parents: microcephaly; paternal grandmother: seizures
11	7	489.60 kb	1,172622–1,662,216	Paternally inherited	*CNTN6*	Exons 3–23 and downstream	DD, ASD, SZs	LD, ADHD, ODD, VSD, feeding difficulties, failure to thrive, brachycephaly, upswept anterior hairline, unusual large hallux, and short second toe	Father: bipolar disorder and ADHD; brother: ADHD, ODD; paternal twin half sisters: DD; mother: bipolar disorder, migraines; maternal grandaunt: migraine
12	9	281 kb	1,189,367–1,470,327	Unknown	*CNTN6*	Exons 4–23 and downstream	LD	No	Father: ID, ADHD, bipolar disorder, depression; mother: bipolar disorder, depression; sibling: LD; sister: ID
13	15	886.76 kb	243,741–1,130,505	Maternally inherited	*CHL1*, *CNTN6*	Whole *CHL1* and exons 1 of *CNTN6*	Obesity, ADHD, bipolar	OCD, migraine, scoliosis, fibromyalgia, rheumatoid arthritis, asthma, irritable bowel syndrome	Mother: migraines, fibromyalgia, psychiatric problem; twin brother and sister: bipolar disorder; maternal half sister: ADHD, psychiatric problems; paternal grandmother: migraines
14	16	2.05 Mb	413,294–2,465,270	Unknown	*CHL1*, *CNTN6*, *CNTN4*	Exons 23–25 of *CHL1*, *CNTN6* and exons 1–2 of *CNTN4*	DD, SZs, dysmorphic	ADHD, OCD, sensorineural hearing loss, asymmetric face, left esotropia, bilateral ptosis, high-arched palate, short stature, single palmar creases, fifth finger clinodactyly, cranial nerve palsy, micropenis	Father: depression; paternal cousin: ASD

*ADHD* attention deficit hyperactivity disorder, *ASD* autism spectrum disorder, *CNV* copy number variation, *DBD* disruptive behavior disorders, *DD* developmental delay, *dup* duplication, *EEG* electroencephalography, *ID* intellectual disability, *LD* learning disability, *NDD* neurodevelopmental disorder, *OCD*, obsessive–compulsive disorder, *ODD* oppositional defiant disorder, *VSD* ventricular septal defect, *SZs* seizures

**Fig. 3:**
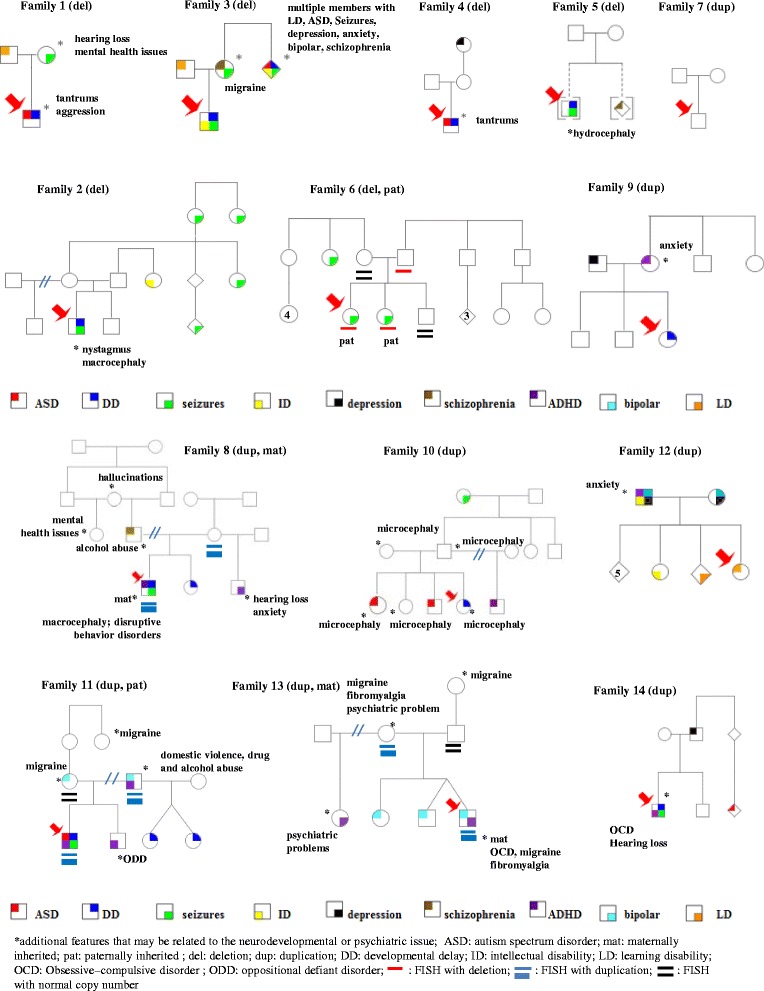
Pedigrees of the 14 families

Thirteen of the 14 patients with these CNVs presented with variable clinical manifestation of neurodevelopmental disorders including ASD in three patients (patients 1, 4, and 11), DD in 10 patients (patients 1–5, 8–11, and 14), SZs in seven patients (patients 2, 3, 5, 6, 8, 11, and 14), and ADHD in four patients (patients 8, 11, 13 and 14). The remaining patient with intragenic deletion of the *CNTN6* was a newborn whose development and behavior at 1 year of age was normal. He was tested because of heart block. In addition, two patients had macrocephaly (patients 2 and 8), one had hydrocephaly (patient 5), and one had microcephaly (patient 10). Other malformations/dysmorphisms were also found in six of the 14 patients, which include craniofacial abnormalities (asymmetric face: patient 14; high-arched palate: patients 2 and 14; schizencephaly: patient 5; brachycephaly: patient 11), digital anomalies (second and third toe syndactyly: patient 10; unusual large hallux and short second toe: patient 11; fifth finger clinodactyly: patient 14), and eye problems (nystagmus: patient 2; esotropia: patient 14; strabismus: patient 3). Family history was available for 13 of the 14 patients, except patient 5 who was adopted. Family 7 had maternal family history of Sjogren’s syndrome. The remaining 12 families had multiple members with NDDs and neuropsychiatric disorders including ADHD, SZs, ASD, ID, schizophrenia, depression, anxiety, learning disability, and bipolar disorder. The pedigree for each family is presented in Fig. 3, and only neurodevelopmental or psychiatric issues are marked in these pedigrees. More detailed family information is summarized in Tables 1 and 2.

## Discussion

Deletions of both *CNTN4* and *CNTN6* genes have been reported in the 3p deletion syndrome, which is characterized by low birth weight, growth restriction, DD, ID, hypotonia, and microcephaly. We present here 13 of the 14 patients with neurodevelopmental disorders and CNVs in the 3p26.3 region encompassing the *CNTN6* gene. The clinical features include ASD, DD, SZs, and ADHD. These CNVs involving the *CNTN6* gene or *CNTN6* and *CHL1* genes have been reported in five patients in three previous independent studies [15–18]. The clinical findings are consistent with the features in our patients. Moreover, macrocephaly presented in two of the 14 patients has been reported in patients with ASD, while microcephaly presented in one of the 14 patients has been reported in chromosome 3p deletion syndrome [21, 22]. Furthermore, microcephaly was also reported in two previously reported patients with deletion of *CNTN6* [16]. In addition to microcephaly, other manifestations of chromosome 3pter-p25 deletion syndrome also exist in one or two of our patients, such as high-arched palate, second and third toe syndactyly, fifth finger clinodactyly, ptosis, joint laxity. and scoliosis (Tables 1 and 2). Syndactyly, clinodactyly and scoliosis, joint laxity and high arched palate were previously reported in patients who have the *CNTN6* deletions [16, 18]. Since the genomic location of the *CNTN6* gene is within the deletion interval for chromosome 3pter-p25 deletion syndrome, losses of the *CNTN6* gene may contribute to the phenotype observed in chromosome 3pter-p25 deletion syndrome. In addition, among the eight patients with gain of copy number in this region five of them had CNV involving partial duplication of the *CNTN6* gene. It is unclear whether the expression of *CNTN6* gene has been altered in these patients as there is a lack of published functional studies of these CNVs. However, partial duplications of other gene, such as partial duplications of the *CHRNA7* gene, have shown to alter the function of the gene product by dominant negative regulation [23]. Unlike 3p terminal deletions which are often de novo events, these *CNTN6* deletions or duplications reported previously and found in our patients are interstitial and are more likely inherited. These deletions and duplications vary in size and in location of breakpoints. The lack of segmental duplications in the surrounding sequences suggests that nonallelic homologous recombination is not the mechanism underlying these deletion and duplication events.

It is striking that 12 of the 13 families have positive family history of various NDDs and neuropsychiatric disorders including ADHD, SZs, ASD, ID, schizophrenia, depression, anxiety, learning disability, and bipolar disorder (Fig. 3; Tables 1 and 2). It is known that the NDDs and psychiatric disorders appear to present a disease spectrum and the outcome of the neurodevelopmental process in each individual patient is determined by interactions among genetic, sociocultural, medical, and environmental factors. Therefore, intrafamilial and interfamilial phenotypic heterogeneity and possible incomplete penetrance are expected. Individual and familial comorbidity among SZs, ASD, bipolar disorder, major depression, ADHD, and other psychiatric diagnoses have been documented by large-scale epidemiological studies [24–27]. These studies suggest a possible genetic overlap between these disorders, which could attribute to the familial vulnerability to NDDs and psychiatric disorders [28]. Risk loci shared by major psychiatric disorders have been reported [29, 30]. Recurrent CNVs in synaptic and neurodevelopmental genes have been found to predispose to a wide spectrum of developmental or psychiatric disorders [30–33]. Similar to other CNVs involving synaptic and neurodevelopmental genes, the CNVs disrupting the *CNTN6/CNTN4* region have also been reported in patients with intellectual disabilities, cognitive disorders, severe anorexia nervosa, and bipolar disorder in addition to ASD [16, 19, 26, 34]. These published records support our hypothesis of variable expression of the CNVs involving the *CNTN6* gene.

Most of the families in our study presented with NDDs and/or psychiatric disorders in both maternal and paternal sides, which makes it difficult to determine the segregation patterns of these CNVs. In family 13, the asymptomatic father has normal copy number for *CNTN6*; the proband with ADHD and bipolar disorder inherited the duplication of *CNTN6* from the mother who has psychiatric problems. In addition, the proband’s maternal half sister has ADHD, and twin brother and full sister have bipolar disorder. However, the copy number for *CNTN6* in these individuals was not determined. In family 6, the proband and her sister with seizures have a paternally inherited deletion of *CNTN6*, and her phenotypically normal mother and brother have normal copy number of *CNTN6*. Although the father of the proband 6 is an asymptomatic carrier of the deletion, it may indicate an incomplete penetrance. Incomplete penetrance was also observed in family 8 (Fig. 3) and in previously reported families [15, 16, 19]. Parental FISH analysis and family history in families 13 and 6 may suggest that the CNVs segregate with the abnormal phenotypes. However, more extended family studies are needed for segregation analysis in the future. Furthermore, most patients have both maternal and paternal family histories of NDDs and/or psychiatric disorders. Therefore, the possibility of a double hit model for inheritance also exists. Based on our study, the *CNTN6* CNVs are rare and presented in about 4 out of 1,000 (14/3,724) patients. At least 40 patients with similar small duplication or deletion involving *CNTN6* gene or *CNTN6* and *CNTN4* genes are reported in the DECIPHER database. These CNVs have also been reported in some phenotypically normal individuals [35]. An expanded CNV morbidity map generated from 29,085 children with developmental delay in comparison to 19,584 healthy controls by Coe et al. [35] showed a frequency of 0.4 % for *CNTN6* CNVs (deletion and duplication) in patients with neurodevelopmental disorders and a frequency of 0.3 % in normal controls. Incomplete penetrance and variable expression are common in neurodevelopmental and neurological disorders. Some patients with these CNVs could have mild or normal phenotype. Therefore, these CNVs can be detected in “healthy individuals.” Finding of the CNVs involving *CNTN6* gene in normal individuals does not exclude the possibility that these CNVs are risk factors for NDDs. Individuals having a mild phenotype may not be recognized.

Unlike other patients, patients 13 and 14 have a CNV involving duplications of *CHL1* and *CNTN6* or *CHL1*, *CNTN6*, and *CNTN 4* genes, respectively. Both patients 13 and 14 have DD, ADHD, oppositional defiant disorder (ODD) and dysmorphic features. In addition, patient 13 has bipolar disorder. Both *CHL1* and *CNTN4* are expressed in the brain and have been proposed as candidate genes for NDDs [1, 36]. Patient 14 has more dysmorphic features including short stature, high-arched palate, bilateral ptosis, clinodactyly, and micropenis, which are commonly present in patient with chromosome 3pter-p25 deletion syndrome. Our study and previous studies found dysmorphic features are also commonly seen in patients who have the CNV involving *CNTN6* gene only [16, 18]. These findings indicate that dosage alterations of *CNTN* genes may affect the normal development of other tissues or organs in addition to the brain.

## Conclusions

We identified 3p26 CNVs involving the deletion/intragenic deletion or duplication/intragenic duplication of *CNTN6* gene in 14 patients. Twelve of the 14 patients have the CNV encompassing *CNTN6* gene alone. Thirteen of the 14 patients have neurodevelopmental behavioral disorders and remarkable family history for neurodevelopment or neuropsychiatric disorders. Seven of the 14 patients presented with dysmorphic features. Our findings provide more evidence to support that deletion or duplication of the *CNTN6* gene is associated with a wide spectrum of neurodevelopmental behavioral disorders. These genotype phenotype correlations pave the way to further investigate the role of *CNTN6* in neurodevelopmental disorders.

## Abbreviations

aCGH: array comparative genomic hybridization
ADHD: attention deficit hyperactivity disorder
ASD: autism spectrum disorder
*CNTNs*: genes for contactin
CNVs: copy number variations
DBD: disruptive behavior disorders
DD: developmental delay
dup: duplication
EEG: electroencephalography
ID: intellectual disability
LD: learning disability
NDD: neurodevelopmental disorder
OCD: obsessive–compulsive disorder
ODD: oppositional defiant disorder
VSD: ventricular septal defect
SZs: seizures
